# Comparison of Physical/Chemical Properties of Prussian Blue Thin Films Prepared by Different Pulse and DC Electrodeposition Methods

**DOI:** 10.3390/ma15248857

**Published:** 2022-12-12

**Authors:** Vahideh Bayzi Isfahani, Ali Arab, João Horta Belo, João Pedro Araújo, Maria Manuela Silva, Bernardo Gonçalves Almeida

**Affiliations:** 1 Centre of Physics of Minho and Porto Universities (CF-UM-UP), LAPMET, Physics Department, University of Minho, Campus of Gualtar, 4710-057 Braga, Portugal; 2 Department of Chemistry and Center of Chemistry, University of Minho, Campus of Gualtar, 4710-057 Braga, Portugal; 3 Faculty of Physics, Semnan University, Semnan P.O. Box 35195-363, Iran; 4 Department of Chemistry, Semnan University, Semnan P.O. Box 35131-19111, Iran; 5 Institute of Physics of Advanced Materials, Nanotechnology and Photonics (IFIMUP), Department of Physics and Astronomy, University of Porto, Rua Campo Alegre, 4169-007 Porto, Portugal

**Keywords:** pulse electrodeposition, Prussian blue, electrochemical devices, electrochemical stability, charge exchange

## Abstract

Prussian Blue (PB) thin films were prepared by DC chronoamperometry (CHA), symmetric pulse, and non-symmetric pulse electrodeposition techniques. The formation of PB was confirmed by infrared spectroscopy (FTIR), energy-dispersive X-ray spectroscopy (EDX) and UV-Vis transmission measurements. X-ray diffraction (XRD) shows the stabilization of the insoluble form of PB. From scanning electron microscopy (SEM) studies, an increase in porosity is obtained for the shorter pulse widths, which tends to improve the total charge exchange and electrochemical stability of the films. While the film prepared by CHA suffered a degradation of 82% after 260 cycles, the degradation reduced to 24% and 34% for the samples prepared by the symmetric and non-symmetric pulse methods, respectively. Additionally, in the non-symmetric pulse film, the improvement in the charge exchange reached ~522% after 260 cycles. According to this study, the deposition time distribution affects the physical/chemical properties of PB films. These results then render pulse electrodeposition methods especially suitable to produce high-quality thin films for electrochemical devices, based on PB.

## 1. Introduction

Prussian Blue (PB) is an inorganic material that has been known since 1703 [1]. It has various applications in different technologies such as electrochromic displays and windows [2,3], batteries [4], energy storage and conversion [5], electrochemical biosensing [6], electrocatalytic systems [7], high-energy-density micro-supercapacitors with ultralow resistance-capacitance (RC) time constants [8] or ultraviolet (UV) photodetectors for smart irradiation monitoring applications [9]. In addition, PB nano-objects have been widely studied and suggested as nano-carriers for drug delivery, as nano-probes for magnetic resonance imaging (MRI) [10], for use in photodynamic/photo-thermal combined cancer treatment [10,11] and for a variety of other applications.

Consequently, in recent years, much research has been devoted to this compound. However, despite the high amount of research on PB and its analogues, there are still some discrepancies in the reported basic properties of PB, such as its crystalline structure or even its chemical composition. For example, according to a great variety of articles [12,13,14,15,16], two different types of PB are reported. They are composed of Fe^III^_4_[Fe^II^(CN)_6_]_3_·m H_2_O and KFe^III^[Fe^II^(CN)_6_]·m H_2_O and are named insoluble and soluble PB, respectively. However, there are also many other articles that introduce the general formula A_1−x_Fe^III^[Fe^II^(CN)_6_]_1−x/4_□_x/4_ for PB [17,18]. In these articles, PB is known as a mixed valence system exhibiting the general formula, in which A is an alkali ion, □ denotes hexacyanoferrate vacancies and x is a stoichiometry parameter [19]. Furthermore, in an extensive study [20], the K_1.9_[Fe^III^_4_Fe^II^_3_ (CN)_18_]·[1.9 OH^−^ + 7.0 H_2_O] chemical formula was proposed for soluble PB and Fe^III^_4_Fe^II^_3_ (CN)_18_·11.0 H_2_O for insoluble PB, which seems to be quite different from the usual formulation. Taking into account these formulations, Fe^III^ and Fe^II^ exist in two rather different stoichiometric groups [21]. However, the common point of all previous research is the composition of the PB backbone. It is based on Fe^II^-C≡N-Fe^III^ chains, and this fact has been accepted by all previous research and for both types of known PB. In addition, the main difference between the two forms of PB arises from the presence of potassium in the soluble form, which is not present in the insoluble form. Another important aspect in the structure of PB is the presence of [Fe^II^(CN)_6_]^4−^ vacancies. Vacancies or large-size [Fe^II^(CN)_6_]^4−^ free ions occur during the PB electrodeposition process. These vacancies have not only been discussed for PB samples [17,21,22]; they have also been reported in many cubic polynuclear transition metal cyanide complexes [23]. F. L. Grandjean et al. [20] presented several images of the unit cell structure of PB ($Pm\bar{3}m$ space group), containing zero, one or two [Fe^II^(CN)_6_]^4−^ vacancies. Based on results obtained by X-ray diffraction, iron K-edge X-ray absorption spectroscopy, pair distribution function analysis, and Mössbauer spectroscopy, the presence of such vacancies was confirmed in insoluble PB as well as K_1.9_[Fe^III^_4_Fe^II^_3_ (CN)_18_]·[1.9 OH^−^ + 7.0 H_2_O] compound [20]. However, this unit cell structure of PB was somehow different from a previously reported for PB [21]. In this report, vacancies were calculated to be around ∼25%, just for insoluble PB that were expected from charge neutrality calculations. 

Furthermore, the presence of water molecules or hydroxyl ions is another fact of the structure of PB. Based on the literature, the water molecules might replace the missing cyanide ligands and become coordinated to the Fe^III^ sites, as normally shown by their oxygen atom. 

Despite the different interpretations in this field, an important result reported in previous work [21,24] is the insertion of potassium ions (K^+^) in the substructure of fresh insoluble PB; this occurs under voltammetric cycling of potassium salt solutions. This process is called the insoluble-to-soluble PB transformation.

The release of the free ions during the voltammetric cycling process should empty the structural vacancies. The vacancy regions are subsequently occupied by a water molecule substructure. X-ray diffraction analyses confirms that this water substructure is formed by two K^+^ ions and six hydroxyl anions after cycling in the potassium salt solution [24]. This final product, which contains K^+^, is known as soluble PB. The Fe^III^_4_[Fe^II^(CN)_6_]_3_·[K^+^_h_·OH^−^_h_·m H_2_O] formula has been suggested for the soluble PB, which is completely different from the KFe^III^[Fe^II^(CN)_6_]·m H_2_O, the former soluble structure proposed in the literature. In this form, there is enough OH^−^ in the water substructure, adjacent to Fe(CN)_6_ vacancies, to compensate for the K^+^ positive ions, forming the K^+^(OH^−^) environment inside the water substructure [21]. In these contexts, the designation between the soluble and insoluble PB refers to the ability of penetration of K^+^ in the substructure of this compound [25]. Nevertheless, the solubility product of the different forms of PB is very low (around 1 × 10^−41^) [26].

Structural changes from insoluble to soluble states have been associated with the partial loss of iron atoms of internal constituent with higher spin and their substitution with K^+^ [24]. However, from the previous discussion and from experimental electrogravimetric analyses, it is observed that there is no loss of high-spin Fe^III^ during the voltammetry cycling process. This leads to a similar crystal structure for both soluble and insoluble PB. Both the soluble and insoluble forms of PB crystallize in the $Pm\bar{3}m$ space group. However, as the concentration of vacancies increases, there is a tendency to change to the $Fm\bar{3}m$ structure, so that when one quarter of the [Fe^II^(CN)_6_]^4−^ anion sites are vacant, and if the vacancies are randomly distributed, the structure can be adequately described in the $Fm\bar{3}m$ space group [20].

The physical or chemical properties of PB are closely related to the water crystalline substructure attached to the main backbone structure, composed of Fe^II^-C≡N-Fe^III^ chains [21], and this depends on the deposition conditions. Additionally, the stabilized structure form is Fe^III^_4_[Fe^II^(CN)_6_]_3_ [21]. This means that the final electrochemically synthesized structure and also the most stable structure is almost similar to the insoluble structure and not to the KFe^III^ [Fe^II^(CN)_6_] structure, as traditionally stated in papers [12,13,14,15,16]. In this respect, the formation of the vacancies, the charge exchange, and the stability of the films are strongly dependent on the deposition methods and conditions.

In most of the previous research, based on PB films, they have been prepared mainly by chemical [27,28] or electrochemical [29,30,31] methods. Spray pyrolysis has also been used in some cases [32]. Electrochemical preparation, in particular, involves different types of methods, including chronoamperometry (CHA) [30,33], chronopotentiometry (CHP) [31,34,35] or cyclic voltammetry (CV) [36,37]. They can also be in the form of DC or pulsed preparation processes [4,38]. 

Pulsed electrodeposition has been used in different forms. The periodical double potential-pulse regime has been used in order to prepare the electroactive composite Prussian Blue/polypyrrole (PB/PPy), from an aqueous medium [39]. Additionally, an optimized pulse electrodeposition protocol has been used to prepare a PB analogue in a porous metallic current collector. This low-temperature technique can then be successfully applied for the preparation of PB, for use as a cathode for Li-ion micro-batteries, with excellent cycling stability [4]. However, there are other possibilities that aim solely at using the pulse technique to control the properties of PB films. In this context, our previous research was focused on the effect of electrodeposition time on the electrochemical behavior and electrochromic properties of PB films, prepared by the DC CHA method [2,33]. On the other hand, here, the present work compares the properties of the films prepared by different methods of pulse and DC CHA, as well as the effect of distribution of pulse width deposition times on the physical/chemical properties of the PB films. Our results indicate that, when using the pulse electrodeposition methods, there is a strong improvement of the PB film’s quality, stability and total charge exchange properties, which are then favorable for their potential application in electrochemical devices.

## 2. Materials and Methods

In this work, PB thin films were prepared by electrodeposition, using the following sequence of steps: first, transparent conducting indium tin oxide (ITO) coated glass plates (Delta Technologies; CG-50IN-1507; 8–12 ohms, Hyderabad, India) were cut with the dimensions of ~1.0 × 3.0 cm^2^ to be used as substrates. Then, the plates were ultrasonically degreased in acetone (Merck, Kenilworth, NJ, USA) and ethanol (Merck), for 10 min, and dried at room temperature. Subsequently, three different PB films were prepared by DC CHA, symmetric pulse, and non-symmetric pulse electrodeposition techniques. The corresponding samples were named PB1, PB2, and PB3, respectively. This procedure was done in a conventional three-electrode cell, containing an Ag/AgCl (saturated KCl) reference electrode, a platinum wire counter electrode, and where the ITO substrate plates were used as working electrodes for film deposition. The electrodeposition solution was prepared using HNO_3_ (100 mM), KNO_3_ (100 mM), Fe(NO_3_)_3_. 9 H_2_O (10 mM), and K_3_[Fe(CN)_6_] (10 mM) [33], in accordance with our previous research. The electrochemical experiments were done with a Potentiostat/Galvanostat (Autolab PGSTAT-12 (Eco Chemie)), at room temperature. Table 1 summarizes the electrodeposition parameters for the prepared films, namely, the reduction voltage (V_R_) and reduction time (T_R_), the oxidation voltage (V_O_) and oxidation time (T_O_) and the number of cycles in pulse electrodeposition. The voltage value for PB electrodeposition was 0.445 V vs. Ag/AgCl, and it was chosen for V_R_ according to our previous research in these materials [33]. The V_O_ value has been set to 0.860 V vs. Ag/AgCl, after its determination as the open circuit potential (OCP) in the described three-electrode cell. The OCP was determined before the beginning of the electrodeposition process. Finally, the electrodeposited samples were rinsed with ultrapure water (Milli-Q Gradient A10 Water Purification System, Millipore Corporation, MA, USA, with a resistivity greater than 18 MΩ.cm^−1^), to remove the excess solution that remained on the PB films. Since the electrodeposition time directly affects the electrochemical, morphological and structural properties of the electrodeposited films, we used the same total reduction time at the end of the cycles (75 s), in all the three samples.

The variation of the current density vs. time and the charge density vs. time were recorded for each sample, during the electrodeposition process.

Fourier transform infrared spectroscopy (FTIR) was performed on the samples, by using a Bruker IFS66V FTIR spectrometer (Billerica, MA, USA) with attenuated total reflection (ATR) mode, using the Golden Gate ATR accessory from Specac.

Morphological analysis of the samples was realized by an ultra-high resolution field emission gun scanning electron microscope (FEG-SEM), NOVA Nano SEM 200, FEI Company (Hillsboro, OR, USA). Secondary electron (SE) images have been obtained at an acceleration voltage 10 kV. In this case, in order to have conductive films, the samples were covered with a very thin film (15 nm) of Au-Pd (80–20 weight %), using a high resolution sputter coater, model 208HR, from Cressington company (Watford, UK), coupled to a MTM-20 Cressington high resolution thickness controller. The histogram of particle diameters was extracted from FEG-SEM images, by using the ImageJ 1.46r image analysis software in an extended area, ~1.7 µm^2^, for each sample.

Microanalysis of the samples was performed to confirm their elemental composition, using the energy dispersive spectroscopy (EDX) technique, with an EDAX Si (Li) detector coupled to the FE-SEM instrument.

X-ray diffraction (XRD) patterns of the PB films were obtained in a Rigaku Smart Lab diffractometer (Tokyo, Japan), with Bragg–Brentano geometry, using Cu–k_α_ radiation (wavelength of λ = 1.5406 Å). The measurements were done in the angular 2θ interval from 10 to 75°, with a 0.01° 2θ step resolution.

A Shimadzu spectrophotometer (Kyoto, Japan), model UV/2501PC, was used to measure the transmittance spectrum and absorption coefficient (α) of each sample, in the wavelength region between 200 to 900 nm.

To study the electrochemical stability of the samples, cyclic voltammetry (CV) was performed for each film, in a solution containing 0.1 M HNO_3_ and 0.1 M KNO_3_, at a scan rate of 100 mV s^−1^. This study was done with the same Potentiostat/Galvanostat model (Autolab PGSTAT-12 (Eco Chemie)) used for the films’ electrodeposition.

## 3. Results and Discussion

### 3.1. Electrodeposition Process

The electrodeposition time has a direct effect on the morphology, thickness, and electrochemical properties of the electrodeposited layers [33]. For example, the layer’s morphology can be strongly affected by changes in the pulse deposition time. This can be due to a variety of factors, namely, the accumulation of the species in the vicinity of the interfaces, the nucleation process and the growth of particles and grains [33]. As such, in order to study the effect of the preparation conditions on the deposition mechanisms, cyclic voltammetry (CV) curves, and SEM micrographs of the PB thin films, electrodeposited under various conditions, were measured.

**Figure 1:**
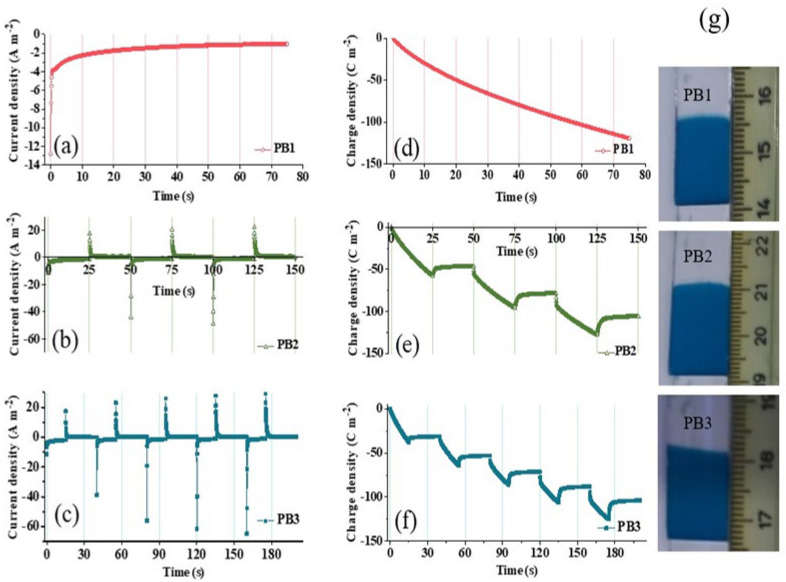
In (**a**–**c**) are the current densities vs. time, measured during the electrodeposition process, in the samples prepared with DC chronoamperometry (PB1), symmetric pulse (PB2), and non-symmetric pulse (PB3) electrodeposition techniques. In (**d**–**f**) are the corresponding charge densities vs. time. In (**g**) is the physical view of the PB samples.

Figure 1a–f shows the current density vs. time and the charge density vs. time, respectively, measured during the electrodeposition process, along with the physical view of each sample (g). The current and charge were normalized to the sample area for film comparison. From the figures it is observed that, when the voltage is applied, there is a fast decrease of the magnitude of the current density, followed by a subsequent stabilization. This behavior is characteristic of the diffusion controlled Cottrell’s behavior [40], where the film deposition depends on the chemical species ability to diffuse to the electrode, where the film is formed. The high initial concentration of the precursor species in the solution around the electrode surface can lead to a large current density at the beginning of each cycle (of the order of some Am^−2^), since its diffusion path to the electrode is small. However, by reducing the available concentration of the species in the vicinity of the electrode, the current density progressively reduces and, finally, it remains almost constant, as shown for sample PB1. In PB2 and PB3 samples, this trend is repeated after each voltage variation, during cathodic half cycles, as observed in Figure 1a–c. As such, the magnitude of deposited charge density increases with increasing time in absolute values in sample PB1, as shown in Figure 1d–f. On the other hand, for the PB2 and PB3 films, the charge density increase also occurs, but in a step-by-step form due to the corresponding variations of the applied voltage.

Notice that, in the case of PB2 and PB3, the sign of the current density has changed from negative to positive when changing the reduction potential to the potential that was assigned to the open circuit potential (0.860 V), as shown in Figure 1a–c. This indicates that, during the assigned open circuit potential phase, the material already electrodeposited in the samples is partially removed from the surface, and is dissolved into the adjacent solution [40]. The de-intercalation of the K^+^ ion under the more positive applied potential also occurs. This effect induces more porosity in the PB2 and PB3 films, as compared to PB1. It also induces different particle sizes and size distributions, as confirmed below by examining the film morphology in the electron microscopy images.

### 3.2. Morphological Analysis

**Figure 2:**
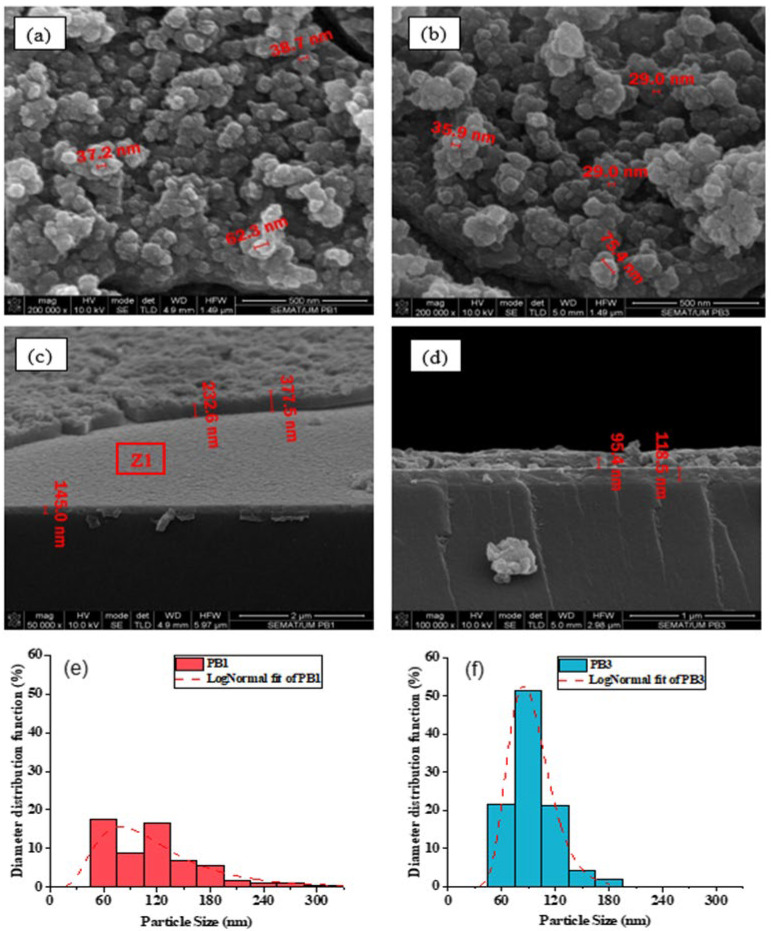
In (**a**,**b**) are the surface and in (**c**,**d**) are the cross-section FE-SEM images of the PB1 and PB3 samples, respectively. In (**e**,**f**) are the corresponding particle’s diameter size distributions. The diameter distributions are fitted with lognormal functions, which are represented by dashed curves.

Surface and cross-sectional views of all the PB films were analyzed by FE-SEM with different magnifications. Figure 2a,b show the surface FE-SEM images of the PB1 and PB3 samples, respectively, while Figure 2c,d show the corresponding cross-section FE-SEM micrographs. Additionally, Figure 2e,f present the particle size distribution histograms of the same samples, estimated by using the ImageJ software.

The surface of the PB films is covered with clusters formed by the agglomeration of nanoparticles. Cracks were observed around the clusters. The particles size distribution was evaluated by fitting the diameter histograms with lognormal functions as shown in Figure 2e,f. The obtained average particle diameters, <x>, have the values of 121 and 94 nm for the PB1 and PB3 films, respectively. The main difference between these samples, which is more obvious from the comparison between Figure 2a,b, is the higher average cluster height and increased porosity in sample PB3 that originates from the distribution of deposition times when using the pulse deposition method. This is more noticeable in PB3 than in PB2 because the electrodeposition process is done in smaller steps in the former, leading to higher porosity of the PB film. Comparing Figure 2e,f shows that PB1 is observed to have a wider range of particle sizes, with higher standard deviations, ω, as compared to PB3. On the other hand, PB3 contains particles with more uniform sizes, which leads to a sharper histogram peak with smaller peak width, ω, in the lognormal fitting. This is in good agreement with the longitudinal growth of clusters in PB3. Figure S1 shows additional cross-sectional SEM images of PB2 and PB3 films at different magnifications, which evidence a slight increase in the surface-to-volume ratio for PB3.

On the other hand, the cross-sectional view of the samples in Figure 2c,d reveals the formation of a compact layer of PB on top of the ITO substrate. Above it, a bumpy surface was formed resulting from the cluster agglomeration, as shown in the surface images of Figure 2a,b. The observed morphology of the PB samples, being clusters formed by the agglomeration of nanoparticles surrounded by cracks, is one of the known morphologies of PB films, as observed in our previous research in this system [33]. However, depending the electrodeposition parameters, and especially the deposition voltage, other morphologies have been reported, such as the formation of cubes and pyramids on the film ‘s surface [41].

### 3.3. Elemental Analysis of the Samples

**Figure 3:**
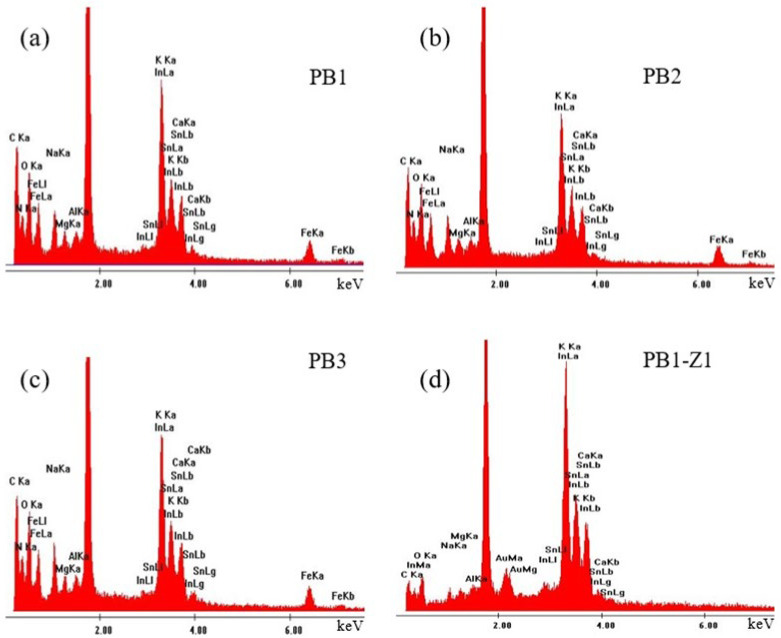
(**a**–**c**) Energy dispersive X-ray spectroscopy (EDX) spectra measured in the PB films deposited over ITO/Glass substrates, using DC chronoamperometry (PB1), symmetric pulse (PB2), and non-symmetric pulse (PB3) electrodeposition techniques. In (**d**), the EDX spectrum of the ITO/Glass substrate is shown, for comparison.

Energy dispersive X-ray spectroscopy (EDX) analysis was performed to study the chemical composition of the samples. The EDX spectra of the deposited PB thin films on top of ITO/Glass substrates are presented in Figure 3a–c. The spectra show the presence of the K, C, N and Fe elements composing the PB films, as well as In, Sn, O, Ca, Na, Mg and Al which are due to the ITO/Glass substrate. The Au peak is also due to the thin layer deposited on top of the sample for the EDX analysis. In order to focus on the characteristics of the PB films, Table 2 contains the atomic percentages (at %) of the elements in the samples, obtained from the spectra of Figure 3. Only the elements related to the PB films have been considered and the results are normalized for the PB composition.

Furthermore, as mentioned in the introduction section and in previous literature [21,24], the main difference between the insoluble and soluble phases of PB is the presence of potassium cations in the water substructures, which fill the vacancies in the PB-soluble form [24]. As a result, the presence of potassium indicates the formation of the soluble phase of PB, along with the insoluble form, during the electrodeposition process. The EDX analysis was also done in the part of the PB1 sample marked by Z1 in Figure 2c. The result is shown in Figure 3d and confirms that it is the surface of the ITO substrate, as expected.

### 3.4. FTIR-ATR Analysis

**Figure 4:**
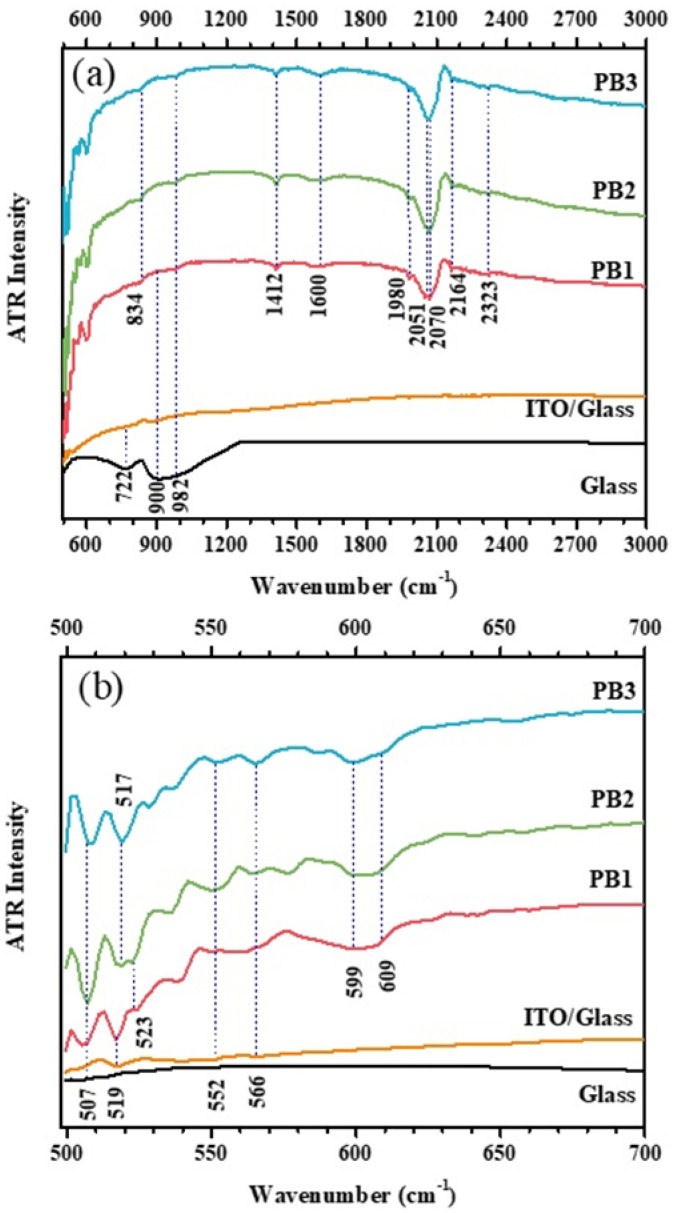
(**a**) FTIR-ATR spectra of the PB films prepared on top of ITO/Glass substrates, measured in the 500–3000 cm^−1^ wavenumber region. In (**b**) is the zoomed spectra in the 500–700 cm^−1^ region. The measured spectra of ITO/Glass and Glass substrates are also shown, for comparison.

To study the formed chemical bonds in the films, FTIR-ATR spectroscopy measurements were performed for wavenumbers between 500 and 3000 cm^−1^, in the mid-infrared region. The analysis was done for all the PB films prepared on ITO/glass substrates, and for ITO/Glass and bare Glass substrates. The measured FTIR-ATR spectra are shown in Figure 4a, while Figure 4b shows a zoomed region. The absorption band at 2064 cm^−1^, which fits well with the Fe^II^-C≡N-Fe^III^ stretching vibration mode in bulk PB [42], has split into two close bands. These are observed at 2051 and 2070 cm^−1^ in our samples, especially in sample PB1. In the literature, the split has previously been observed during ex situ external reflectance investigations of electrodes, modified with oxidized indium and palladium hexacyanoferrates [43,44]. The peak at 2051 cm^−1^ is due to the CN bond of PB as mentioned above.

Although the exact assignment of the peak at 2070 cm^−1^ is not made, it is probably due to the CN bond belonging to Fe^II^-C≡N⋯Fe^III^⋯OH_2_ or Fe^II^-C≡N⋯Fe^III^⋯OH^−^ [45]. Also, the band appearing at 2164 cm^−1^ is attributed to cyanide band splitting, which accompanies oxidation. The peak splitting may suggest strong cyanide bridging between two equivalent Fe^III^ ions within the Fe^II^-C≡N-Fe^III^ units of PB.

The broad band beyond 2400 cm^−1^ can refer to the surface absorbed water molecules and the O-H stretching vibrations of hydroxyl groups [46]. Furthermore, the absorption band around 1600 cm^−1^, due to the H-O-H bending vibration mode, is another confirmation of the presence of interstitial water substructures inside the PB thin films. This means that water molecules do not re-coordinate with iron ions but are incorporated into interstitial positions in the PB lattice. The peak at 1412 cm^−1^ arises from the O-H bending mode.

Some of the bands below 1200 cm^−1^ are characteristic of the Glass and ITO/Glass substrates. Namely, the peaks at 982, 900, 722 and 507 cm^−1^ are from the glass, while the low wavenumber bands at around 566, 552 and 519 cm^−1^ are due to the indium–oxygen bonds in ITO [47,48].

The peaks around 599 and 609 cm^−1^ are from the metal–oxygen bond vibration bands, since the characteristic metal–oxygen bond formation is observed in the region of 400–850 cm^−1^ [49]. One NO_3_^−^ vibration appears around 834 cm^−1^ [47]. This can refer to the precipitation of a small amount of the NO_3_^−^ precursor which also explains the observed amount of nitrogen and oxygen in Table 2.

The band around 500 cm^−1^ is also due the structure of Fe^II^-C≡N-Fe^III^ linkage of PB. This peak is related to the presence of coprecipitated ferricyanide ions [50,51].

### 3.5. Structural Analysisof the PB Films

**Figure 5:**
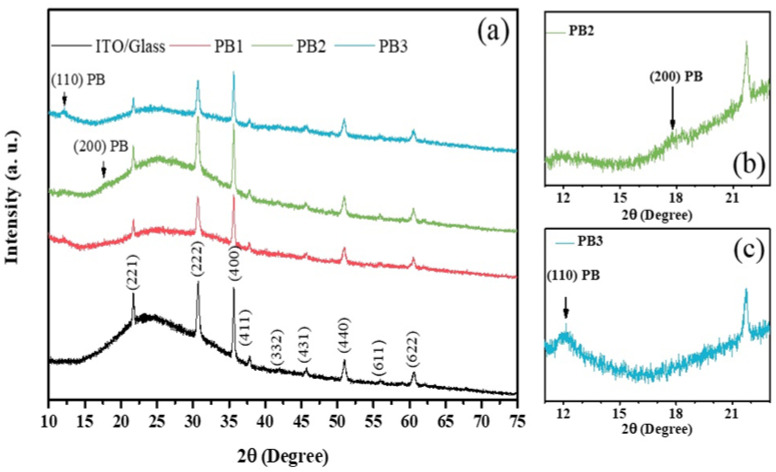
X-ray diffraction (XRD) patterns of the (**a**) PB films prepared over ITO/Glass substrates, along with the corresponding diffractogram obtained in an ITO/Glass sample. In (**b**,**c**) are the XRD patterns for samples (**b**) PB2 and (**c**) PB3.

Figure 5a shows the X-ray diffraction (XRD) curves measured in the PB films along with its ITO/Glass substrate, for 2θ angles between 10° and 75°. The main observed peaks are due to the ITO substrate and the bulge observed in all patterns, with a maximum around 24°, is due to the amorphous nature of the glass substrate. However, according to Figure 5b,c, a peak is observed at an angle of ~12.1° and a shoulder appears at around 17.9°, which are due to the PB films. They correspond to the (110) and (200) lattice planes of the cubic $Pm\bar{3}m$ structure of PB, both in the soluble and insoluble phases [20]. The (110) peak was fitted with a Gaussian function to determine its position and full width at half maximum. The lattice parameters determined from the (110) peak positions were 1.036 nm, 1.059 nm, and 1.035 nm for the PB1, PB2, and PB3 films, respectively. For bulk PB the lattice parameter is *a* = 1.02178 nm for the insoluble form and *a* = 1.02059 nm for the soluble one [20]. In this respect, the lattice parameters of the films are closer to the corresponding value in the insoluble form. Additionally, the intensity of the (110) peak is one order of magnitude higher in the insoluble form of bulk PB, as compared to the soluble one, and, thus, more prone to be seen in XRD, as observed below.

As such, the XRD structural results indicate that the films present mainly the insoluble phase of PB. The grain sizes (*L*) were estimated from the *FWHM* of the (110) peak using the Scherrer equation, Equation (1) [52,53,54]:

(1) $$L=\frac{0.94\lambda }{FWHM\cdot \cos\left(\theta \right)}$$

where *λ* is the X-ray wavelength and *θ* is the Bragg diffraction angle. The calculated grain size values are in the range of 11–20 nm, which are slightly lower, but in agreement with the particle size determined from the SEM results in Figure 2a,b. Thus, the XRD analysis performed in our samples indicates that they are composed mainly by the insoluble form of PB, which is in agreement with the obtained SEM/EDX results. The low intensity of the PB peaks in the XRD spectra is mainly due to the small thickness of the films in the studied samples.

### 3.6. UV-Vis Spectroscopic Analysis

**Figure 6:**
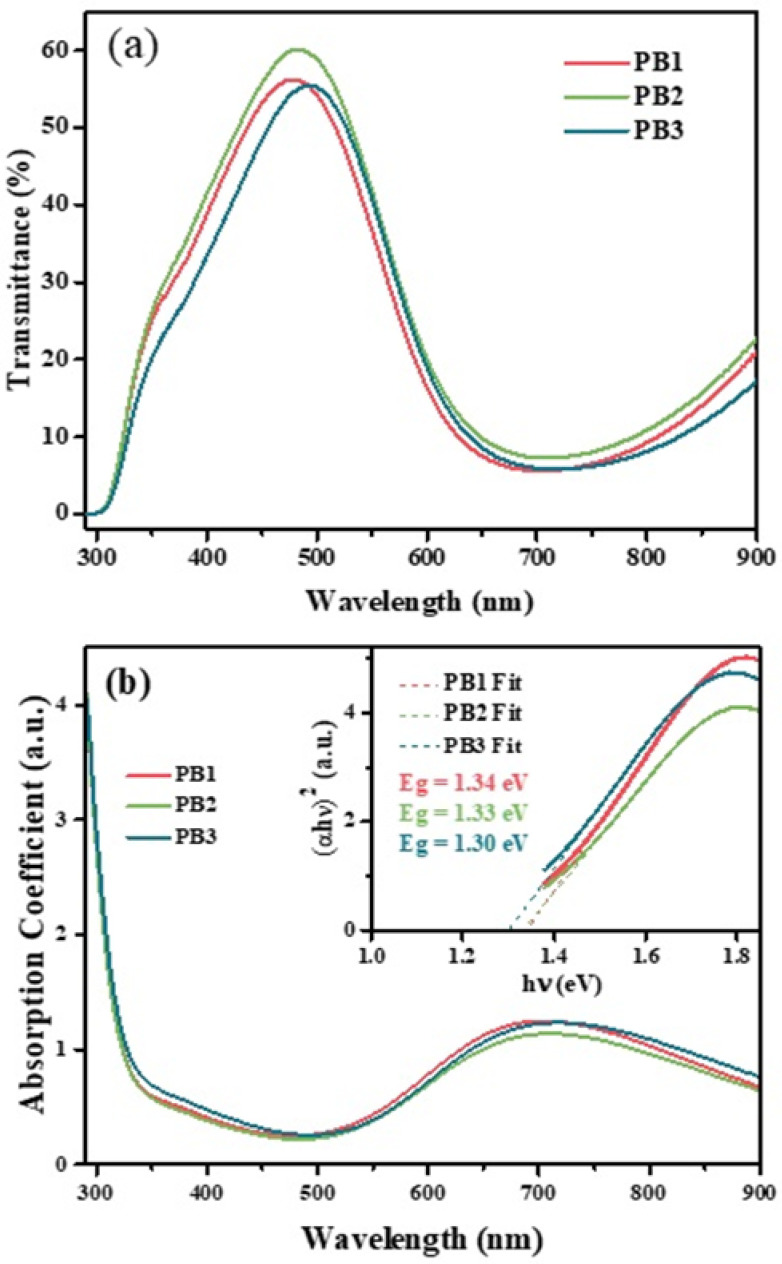
(**a**) Transmittance spectra and (**b**) absorption coefficient (α) of the PB films. The inset of (**b**) shows the Tauc plot for the bandgap determination.

The transmittance spectra of the PB films, recorded between 200 and 900 nm, are presented in Figure 6a. A sharp peak was observed in the transmittance spectra of the films. It occurs at the wavelengths of 478, 483, and 493 nm for the PB1, PB2, and PB3 films, respectively, with transmittances of 56.2, 60.1 and 55.5%. The corresponding absorption coefficient, α, is also shown in Figure 6b.

The strong increase of the absorption for wavelengths below 300 nm is due to the ITO layer on the glass substrate [55]. Additionally, an absorption band is observed, with a peak at the wavelengths near 704, 708, and 718 nm for PB1, PB2, and PB3, respectively. This peak is associated with the intervalent charge transfer of an electron from Fe^II^ to Fe^III^ ions, through the Fe^II^_low spin_-CN-Fe^III^_high spin_ pathway [56]. It indicates a strong absorption in the red by the films, giving them a preferential blue color, which is characteristic of PB [20,24,56,57] (Figure 1).

The absorption coefficient, *α*, in the high-absorption region, is given by the Tauc equation, Equation (2) [58,59], as:

(2) $$\alpha =\frac{A{\left(h\upsilon -E_{g}\right)}^{1/2}}{h\upsilon }$$

where *A* is a proportionality constant that depends on the transition probability, *hν* is the photon energy and *E_g_* is the optical bandgap. By plotting ${\left(\alpha h\upsilon \right)}^{2}$ as a function of the energy, hυ, a linear dependence is obtained. *E_g_* is calculated by extrapolating the corresponding line to α=0. The inset of Figure 6b shows the Tauc plot used in order to determine the band gap energies, *E_g_*, of the PB films. The obtained values are 1.34, 1.33, and 1.30 eV for PB1, PB2, and PB3, respectively. These values are slightly lower than for bulk PB, where *E_g_* = 1.75 eV [56]. The decreasing value of *E_g_* from PB1 to PB3 can be related to the multi-step deposition process, with progressively shorter pulse deposition times, that affects the morphology and arrangement of particles during the sample electrodeposition.

### 3.7. Cyclic Voltammetry Analysis

**Figure 7:**
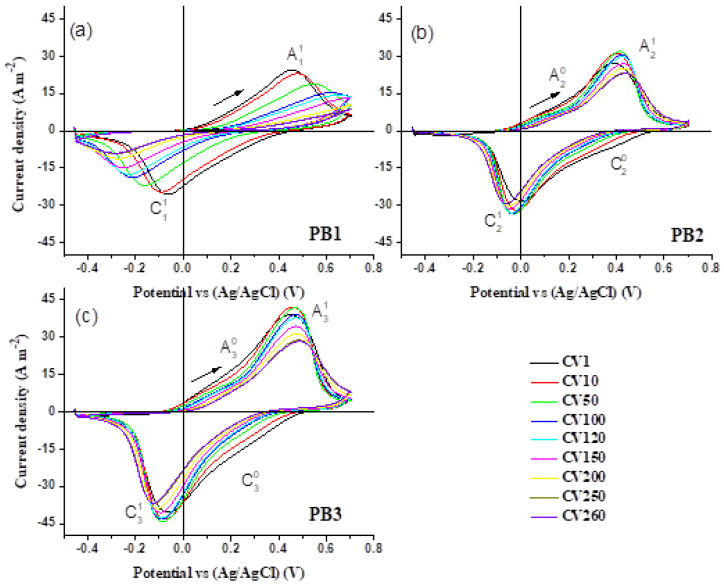
Cyclic voltammograms of the (**a**) PB1, (**b**) PB2 and (**c**) PB3 films, recorded in a solution containing 0.1 M HNO_3_ and 0.1 M KNO_3_, at a scan rate of 100 mVs^−1^.

The electrochemical performance of the PB films was evaluated by cyclic voltammetry (CV). Figure 7 presents the cyclic voltammograms of the electrodeposited PB films. The analysis was performed between −0.455 V and 0.700 V and was started from −0.445 V in the anodic direction. The results were recorded for at least 260 continuous cycles for each PB film. They were obtained in a three-electrode cell, with the PB film acting as working electrode, a Pt wire as the counter electrode and Ag/AgCl as the reference electrode. A solution of HNO_3_ (0.1 M) and KNO_3_ (0.1 M) was used as an electrolyte and the studies were done at a scan rate of 100 mVs^−1^.

Several parameters influence the form of the CV curves. The most important ones are the number of electrodes and the type of electrochemical cell, the voltage region, the scan rate and the electrolyte [60].

According to Figure 7a–c, two characteristic peaks are visible in the voltammograms. They are called $A_{i}^{1}$ and $C_{i}^{1}$, for samples i= 1, 2 and 3, These peaks are expected for PB and they correspond to the conversion of the compound between its blue and bleach states, respectively. They are in agreement with previous reports [33,61], with the slight shifts in peak positions originating from the difference of solutions and other cell parameters. In addition, reproducible curves were generally obtained for the cycled PB films, as cations move into and out of the PB lattice on negative and positive scans, respectively. However, there are some differences between the CV results for PB1, seen in Figure 7a, as compared to the other samples. In the case of PB1, the reduction and oxidation peaks shift to more negative and positive potentials, respectively, under cycling. On the other hand, for PB2 and PB3, the peak positions remain nearly constant, as presented in Table S1 of the Supplementary Materials. Additionally, the area enclosed in the CVs was significantly reduced for the PB1 sample upon cycling, while in samples PB2 and PB3 prepared by the pulse method, the CV areas did not change significantly. This is a manifestation of a reasonable reversibility for PB2 and PB3.

The electrochemical stability of the PB films was analyzed by determining the percentage of degradation, *χ*, according to Equation (3), in which *Q_m_* is the exchange charge density during the *m*th CV cycle.

(3) $$\chi \left(\%\right)=\frac{Q_{n}-Q_{m}}{Q_{n}}\times \;100\%,\;n\;=\;\mathrm{Reference number and}\;m\;=\;\mathrm{CV cycle number}$$

Based on previous reports [60,61,62], the degree of degradation of electrochemical systems is obtained by comparing the exchange charge between the *m*th CV and reference cycle, which is generally the second CV cycle. In this study, we evaluated the degradation degree of the prepared PB films after 260 cycles. However, for a clear understanding of the system degradation, we compared this quantity in two ways: in the first case, the second CV was used as reference, while in the second case, the tenth CV was used as reference. The above parameters are referred as R2 and R10 in Table 3. This comparison is important, since in the first 10 cycles the CV areas show an increase, which decrease upon subsequent cycling.

This initial area enhancement is related to the different sample morphologies, leading to different charge exchange capacities through the films, as discussed above. In addition, comparing the degradation degree by considering the second and tenth cycles as two different references, allows estimating the maximum degree of degradation.

The area of the CV curves was calculated during the anodic and cathodic scans, which are proportional to the anodic and cathodic exchange charge densities in each cycle. These parameters are presented in Table 3 as Q_Anodic_ and Q_Cathodic_, respectively. Total exchange charge densities, Q_Total_, were also calculated from the obtained Q_Anodic_ and Q_Cathodic_ and they are presented in Table 3. Based on these results, PB3 presents a higher total exchange charge, which varies from 291 C m^−2^ to 192 C m^−2^ after 260 cycles. In addition, Q_Total_ shows an improvement from sample PB1 to PB3, which corresponds to progressively shorter pulse deposition times. The obtained Q_Total_ values on the second CV were 169 C m^−2^, 189 C m^−2^ and 291 C m^−2^, for PB1, PB2, and PB3, respectively. This improvement in the exchange charge, from PB1 to PB3, reaches ~522 % after 260 cycles.

Considering the second CV as the reference, the degradation degree of PB1 is 82%, as shown in Table 3. However, this parameter reduced to 24% and 34% for PB2 and PB3, respectively. These values change to 81%, 23%, and 30% when using the 10th CV as reference.

**Figure 8:**
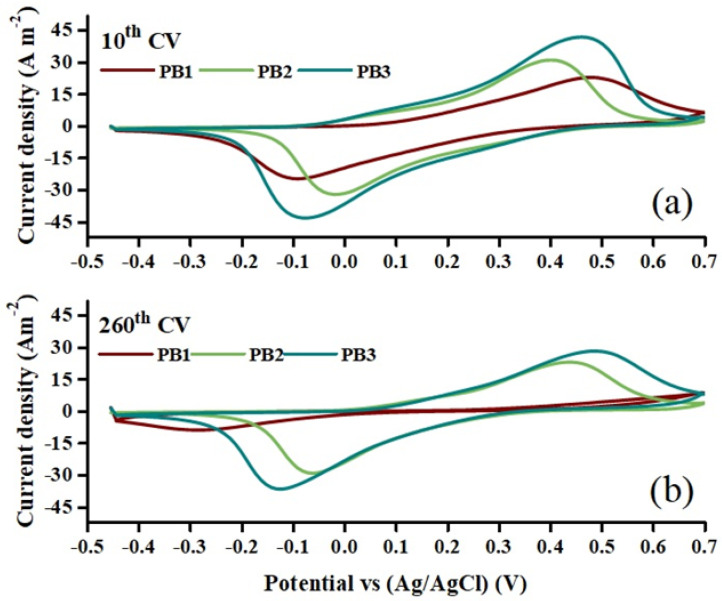
Comparison of the (**a**) 10th and (**b**) 260th cyclic voltammograms of the PB films, recorded in a solution containing 0.1 M HNO_3_ and 0.1 M KNO_3_, at scan rate of 100 mV s^−1^.

Figure 8a,b presents the comparison of the 10th and 260th CVs of the PB films. The figure clearly confirms a larger CV area and a higher amount of exchange charge for the samples prepared by pulse methods. These results indicate a better electrochemical activity for the PB films prepared by the pulse method in comparison with the one prepared by the DC method. According to Figure 8, the CV of PB1 collapses after 260 cycles, which is not seen in the case of PB2 and PB3 films. The greater stability of the films prepared by the pulse method makes these films promising for application in electrochromic devices, sensors, displays and other electrochemical devices. On the other hand, separating the deposition time in shorter pulse intervals (15 s for PB3, as compared to 25 s for PB2), leads to a more porous layer in PB3. This then tends to increase the ion exchange and causes a larger area of CVs for PB3, at the expense of a slightly higher degradation.

Additionally, while PB1 experiences only one pair of redox peaks in each CV cycle, in the interval of voltage under study (Figure 7a), the two other samples prepared by the pulse method undergo more redox reactions (Figure 7b,c). These are presented as $A_{2}^{0}$ and $A_{3}^{0}$, during the anodic half cycle and $C_{2}^{0}$ and $C_{3}^{0}$, during the cathodic half cycle, in Figure 7. This indicates extra intermediate oxidation products, which appear as redox shoulders at ~0.2 V during the anodic and cathodic scanning.

Considering the initial predominant composition of the films as insoluble PB, starting the CV cycling from −0.445 V leads to a prompt reduction to Prussian White (PW), with the chemical formula K_4_Fe_4_^II^[Fe^II^(CN)_6_]_3_, with interstitial water [45]. In the anodic scanning, the $A_{2}^{0}$ and $A_{3}^{0}$ shoulders, which are more evident under cycling, may originate from releasing the interstitial water [21] accompanied by the oxidation of PW to PB. A possible complementary process is the incorporation of interstitial water, as well as K^+^ ions, manifested in the form of $C_{2}^{0}$ and $C_{3}^{0}$ cathodic shoulders that were followed by the reduction of PB.

Continuing the voltammetric cycling in the vicinity of an electrolyte containing K^+^ ions, the potassium ion penetrates the insoluble PB substructure, which leads to soluble PB. The difference between the soluble and insoluble structures lies in the occupation fraction of the ionic entities in the water substructure, as it has been clarified in the literature [21,63]. The structural analysis by P. R. Bueno et al. [21] on the stabilized PB compounds after different CV cycles indicates a Fe^III^_4_ [Fe^II^ (CN)_6_]_3_·[K^+h^ ·OH^-h^·m H_2_O] structure, very similar to that proposed by Herren et al. [63] as Fe^III^_4_[Fe^II^(CN)_6_]_3_ ·m H_2_O. The stabilized soluble PB may then convert to another bleached state known as Everit Salt (ES). As the CV cycle progresses, the reversible reoxidation of ES to PB and vice versa continues [64]. $K_{2}{\mathrm{Fe}}^{\mathrm{II}}\left[{\mathrm{Fe}}^{\mathrm{II}}{\left(\mathrm{CN}\right)}_{6}\right]$ with interstitial water is a possible formula for ES [37,64]. In this context, the distribution of deposition times in the samples prepared by pulse methods affects the morphology of the films, enhancing the surface to volume ratio and creating a larger surface area. This facilitates the exchange of water to the film structure, which then leads to the observed shoulders in the CV, as shown in Figure 7.

These types of CVs with the large peak-to-peak potential separation have been reported in previous researches [33], however, in order to further clarify the origin of the large peak-to-peak potential separation in the CVs, scans at different potential scan could be performed [65,66].

## 4. Conclusions

Because the ease of charge exchange and electrochemical stability are some of the most important parameters in the manufacture of electrochemical devices, such as electrochromic windows, sensors, displays, batteries, etc., in this work the effect of electrodeposition time distribution on the physical and chemical properties of PB films was studied. For this purpose, PB films were prepared by DC CHA, symmetric pulse, and non-symmetric pulse electrodeposition techniques, over ITO/Glass substrates. FTIR, EDX and UV-Vis spectroscopies, along with X-ray diffraction measurements, have shown the formation of PB films with the insoluble structure. From the SEM results, it was observed that the division of electrodeposition times into discrete and shorter time intervals, in samples prepared by the pulse method, can effectively affect the morphology of the films. In pulsed samples, multistep deposition under periodic applied voltages leads to the formation of smaller particles and less compact and more porous films that clearly facilitate charge exchange. In this regard, the samples prepared by the pulse method have much higher stability and much lower degradation compared with those prepared by the DC CHA method. Additionally, for the samples prepared with the non-symmetric pulse electrodeposition, the total exchange charge, Q_Total_, improved by up to ~522%, compared to the DC method, after 260 cycles. This improved stability along with an improved total exchange charge makes the pulse electrodeposition methods particularly suitable for the preparation of PB thin films, for application in electrochemical devices.

## Figures and Tables

**Table 1:** PB thin film electrodeposition parameters, namely: reduction voltage (V_R_), oxidation voltage (V_O_), reduction time (T_R_), oxidation time (T_O_), and number of cycles for pulse electrodeposition.

Sample Name	PB1		PB2					PB3				
Method	DC CHA		Symmetric Pulse					Non-Symmetric Pulse				
Parameters	V_R_ (V)	T_R_ (s)	V_R_ (V)	T_R_ (s)	V_O_ (V)	T_O_ (s)	Cycles Number	V_R_ (V)	T_R_ (s)	V_O_ (V)	T_O_ (s)	Cycles Number
Values	0.445	75	0.445	25	0.860	25	3	0.445	15	0.860	25	5

**Table 2:** The normalized data regarding the EDX spectra of the PB films of Figure 3.

EDX	C (at %)	N (at %)	O (at %)	K (at %)	Fe (at %)
PB1	28.22	33.12	30.40	3.78	4.48
PB2	27.23	34.49	29.87	3.39	5.01
PB3	26.76	34.45	30.96	3.36	4.46

**Table 3:** The values of anodic, cathodic, and total exchange charges regarding the 2nd, 10th, and 260th CVs for all the PB films, prepared by DC (PB1), symmetric (PB2) and non-symmetric (PB3) pulse methods. R2 and R10 refer to the 2nd and 10th CV reference cycles, respectively.

Sample	PB1			PB2			PB3		
Parameters	Q_Anodic_ (C m^−2^)	Q_Cathodic_ (C m^−2^)		Q_Anodic_ (C m^−2^)	Q_Cathodic_ (C m^−2^)		Q_Anodic_ (C m^−2^)	Q_Cathodic_ (C m^−2^)	
Cycle Number									
2	81	87		89	100		139	152	
10	77	81		92	96		134	141	
260	14	17		71	73		93	99	
**Parameters**	**Q_Total_** **(C m^−2^)**	**χ (%)**		**Q_Total_** **(C m^−2^)**	**χ (%)**		**Q_Total_** **(C m^−2^)**	**χ (%)**	
**Cycle** **Number**		**R2**	**R10**		**R2**	**R0**		**R2**	**R0**
2	169	82	81	189	24	23	291	39	30
10	158			187			274		
260	31			144			192		

## Data Availability

Not applicable.

## Supplementary Materials

The following supporting information can be downloaded at: https://www.mdpi.com/article/10.3390/ma15248857/s1, Table S1: The values of anodic/cathodic peak current densities (j_p_) and potential (E_p_) values for PB films according to Figure 7. Figure S1: The cross-sectional SEM images of PB2 and PB3 films at different magnifications.
