# Supplementary material for: Comparison of Physical/Chemical Properties of Prussian Blue Thin Films Prepared by Different Pulse and DC Electrodeposition Methods
Source: Materials (Basel). 2022 Dec 12;15(24):8857. doi: 10.3390/ma15248857 (PMC9782874; doi:10.3390/ma15248857)
Supplement: Supplementary file 1 [file materials-15-08857-s001.zip › materials-1996433-supplementary.pdf]

materials

MDPI

Supplementary Information

Comparison of physical/chemical properties of Prussian Blue thin films prepared by different Pulse and DC electrodeposition methods

Vahideh Bayzi Isfahani [1,2,3,*], Ali Arab [4], João Horta Belo [5], João Pedro Araújo [5], Maria Manuela Silva [2] and Bernardo Gonçalves Almeida [1,*]

1 Centre of Physics of Minho and Porto Universities (CF-UM-UP), LAPMET, Physics Department, University of Minho, Campus of Gualtar, 4710-057 Braga, Portugal
2 Department of Chemistry and Center of Chemistry, University of Minho, Campus of Gualtar, 4710-057 Braga, Portugal
3 Faculty of Physics, Semnan University, Semnan P.O. Box 35195-363, Iran
4 Department of Chemistry, Semnan University, Semnan P.O. Box 35131-19111, Iran
5 Institute of Physics of Advanced Materials, Nanotechnology and Photonics (IFIMUP), Department of Physics and Astronomy, University of Porto, Rua Campo Alegre, 4169-007 Porto, Portugal
* Correspondence: v.b.isfahani@gmail.com (V.B.I.); bernardo@fisica.uminho.pt (B.G.A.)

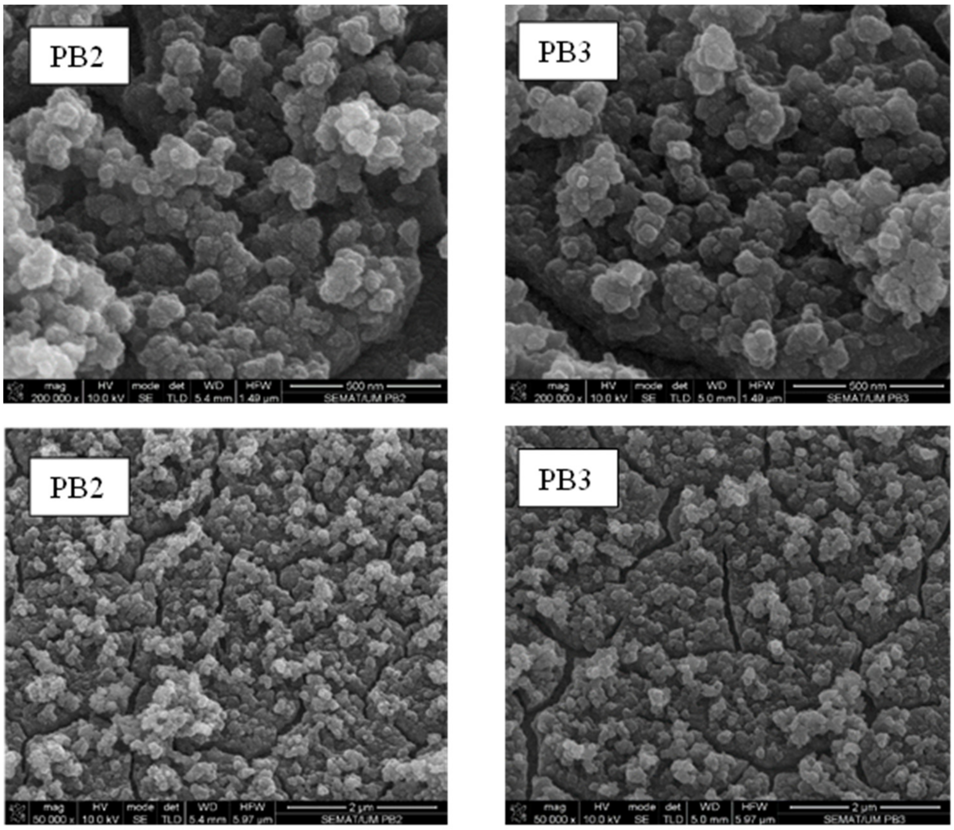

Figure S1. The cross-sectional SEM images of PB2 and PB3 films at different magnifications.

Table S1. The values of anodic/cathodic peak current densities (jp) and potential (Ep) values for PB films according to Figure 7. Some of the anodic peaks are out of studied region.

**Parameters**		**Cathodic**								
Sample	Cycle	1	10	50	100	120	150	200	250	260
PB1	E_p (V)	-0.066	-0.094	-0.162	-0.213	-0.227	-0.251	-0.269	-0.284	-0.287
	j_p (A m$_{-2}$)	-25.6	-24.7	-22.0	-18.8	-17.6	-14.8	-11.2	-9.3	-8.9
PB2	Ep (V)	0.001	-0.017	-0.031	-0.037	-0.041	-0.046	-0.053	-0.062	-0.063
	i_p (A m$_{-2}$)	-28.3	-32.0	-33.5	-33.3	-33.0	-31.4	-30.5	-29.5	-29.3
PB3	E_p (V)	-0.063	-0.077	-0.087	-0.091	-0.094	-0.094	-0.106	-0.123	-0.126
	j_p (A m$_{-2}$)	-40.4	-43.1	-44.2	-43.3	-42.8	-40.6	-38.8	-37.1	-36.7
**Parameters**		**Anodic**								
Sample	Cycle	1	10	50	100	120	150	200	250	260
PB1	E_p (V)	0.456	0.477	0.549	0.623	0.652	-		-	-
	j_p (A m$_{-2}$)	24.3	23.0	18.7	15.2	14.3	13.3	-	-	-
PB2	E_p (V)	0.39	0.401	0.418	0.425	0.426	0.431	0.434	0.435	0.437
	j_p (A m$_{-2}$)	27.1	31.2	31.9	30.4	29.7	27.0	25.0	23.3	23.0
PB3	Ep (V)	0.449	0.462	0.471	0.473	0.474	0.474	0.478	0.485	0.487
	j_p (A m$_{-2}$)	39.0	42.0	41.7	39.0	37.8	34.3	31.4	28.8	28.2
